# Effects of Polysaccharides From *Auricularia auricula* on the Immuno-Stimulatory Activity and Gut Microbiota in Immunosuppressed Mice Induced by Cyclophosphamide

**DOI:** 10.3389/fimmu.2020.595700

**Published:** 2020-11-06

**Authors:** Xianghui Kong, Weiwen Duan, Dingjin Li, Xiaoxian Tang, Zhenhua Duan

**Affiliations:** ^1^ Institute of Microbiology, Heilongjiang Academy of Sciences, Harbin, China; ^2^ Institute of Food Research, Hezhou University, Hezhou, China

**Keywords:** *Auricularia auricula*, polysaccharides, immune-stimulatory activity, gut microbiota, short-chain fatty acid

## Abstract

Recently, the immuno-enhancing potential of polysaccharide from *Auricularia auricula* (AAP) has been an area of research interest. However, the immune-stimulatory activity and mechanisms of AAP in immunosuppressive mice treated with cyclophosphamide (CTX) are still poorly understood. This study aimed to evaluate the immuno-enhancing effects of AAP and mine its possible mechanisms. Firstly, polysaccharides were isolated from *A. auricula* and purified. Secondly, the immune-stimulatory activities of the first AAP fraction (AAP1) were evaluated in the CTX-treated mice. Results showed that AAP1 significantly enhanced immune organ indexes, remarkably stimulated IFN-γ, IL-2, IL-4, IL-10, and TNF-α levels in the serum, and dramatically up-regulated the mRNA levels of Claudin-1, Occludin and ZO-1. Compared to the CTX group, AAP1 administration restored the gut microbiota composition similar to that of the control group by decreasing the ratio of Firmicutes/Bacteroidetes and increasing the relative abundances of short-chain fatty acid-producing microbiota. This study provides useful information for its further application as an immune-stimulator in foods and drugs.

## Introduction

Chemotherapy is widely recognized as a therapeutic procedure for treating tumor growth (1). Among the agents, it often involves the use of cyclophosphamide (CTX), reputed for treating a variety of cancers and autoimmune disorders (2–4). Despite of these crucial functionalities, demerits in CTX usage, including adversely altering gut microbiota modulation and mucosal barrier breakdown, severely disrupts healthy intestinal composition (5, 6). Consequently, research into the possible replacement of CTX by discovering immune-friendly agents that would not only lower the side effects of chemotherapy but also increase its curative potentials, has intensified over the years.

The human gut microbiota is a complex ecosystem made up of trillions of microbes that perform an array of functions from the gastrointestinal tract. Indeed, it is safe to say that the ability of the gut microbiota to adapt to the host’s needs through several immune and metabolic pathways largely determines host health status and response to diseases (7). Accumulating evidence support the postulation that the complex gut ecosystem has a strong relationship with the proper development of the host immune system (8, 9). The latter, aside from ensuring the detection and removal of harmful organisms, also prevents the dysbiosis of the gut microbiota, suggesting a complex beneficial interaction. Imbalances in the gut microbiota have been implicated in many immune and immune-related disorders (10).

Gut health and host immunity improvement using natural polysaccharides have been recently reported, and the findings therein have opened new frontiers in the application of functional foods and pharmaceutics. Many of these natural polysaccharides are non-digestible, allowing them to transit the upper gastrointestinal tract and undergo fermentation by an array of gut microbes in the caecum and colon. On the one hand, natural non-digestible polysaccharides are favorable substrates to improve the intestinal ecosystem by promoting the growth of health-promoting microorganisms and reducing the abundance of opportunistic pathogens. The polysaccharides from purple sweet potato could regulate the gut microbiota by increasing the abundance of Bacteroidetes and decreasing the abundances of Firmicutes and Proteobacteria (11). The polysaccharides from *Codonopsis pilosula* were able to elevate the number of *Lactobacillus* and lower the number of *Escherichia coli* in the cecum (12). On the other hand, natural polysaccharides may improve several immune responses by promoting microbial immune-regulatory molecules. The polysaccharides from *Lycium barbarum* and *Caulerpa lentillifera* could enhance the production of short-chain fatty acids (SCFAs) in the colon for immunoregulation (13, 14). In addition, squid ink polysaccharides could promote intestinal secretory immunoglobulin A secretion to ameliorate the immune disorders (15, 16).

*Auricularia auricula*, one of the colloid fungus, has been widely used as medicine and food, particularly in East Asian countries like China and Korea (17). Its high nutritive value is based on its high carbohydrate, amino acids, trace elements and vitamins content. This fungus has also been included in many foods consumed in the countries mentioned above (18). In recent years, *A. auricula* polysaccharides (AAP) have been suggested to play important biological roles, such as hypoglycemic, hypolipidemic, antioxidant, antitumor and antiviral activities (17, 19–24). Although, the immuno-enhancing potential of AAP has attracted research interests (25–27). the immuno-enhancing mechanisms of AAP *in vivo* from the insight of gut microbiota are still poorly understood. Therefore, this study aimed to evaluate the immuno-enhancing effects of AAP on immune organ indexes, cytokine production, and the gut microbiota composition in immunosuppressive mice treated with CTX and mine its possible mechanisms. This study provides useful information for the application of AAP in immunoregulation.

## Materials and Methods

### Materials and Chemicals

*A. auricular* was obtained from the edible fungus base in Heilongjiang province. Standards of acetate, propionate, and butyrate were obtained from Aladdin Chemical Reagent Co., Ltd (Shanghai, China). CTX was obtained from Shanghai Ryon Biological Technology Co., Ltd (Shanghai, China). All other reagents used in this study were purchased from Sinopharm Chemical Reagent Co., Ltd (Shanghai, China) and were of analytical grade.

### Isolation and Purification of AAP

AAP was extracted as described by Zeng et al. (21). with slight modifications. Briefly, *A. auricular* powder (20 g) was extracted with 1000 mL deionized water for 2 h at 90°C. The total extraction solution was concentrated under reduced pressure to 1/3 of the original volume and then precipitated with three volumes of chilled 95% ethanol at 4°C overnight. Protein content was removed by applying the Sevag reagent (chloroform : *n*-butyl alcohol = 4:1, v/v) to the precipitate solution. The aqueous solution was dialyzed against deionized water for 3 days (7 kD), and the water was changed three times a day. Crude polysaccharides were obtained by concentrating and freeze-drying the resultant solution. Purification was carried out using an anion exchange chromatography on a DEAE-52 (GE Healthcare Life Sciences, USA) column (ID 20 mm × 50 cm) as described by Han et al. (20) Four fractions (AAP1, AAP2, AAP3, and AAP4) were obtained. A Sephacryl S-400 (GE Healthcare Life Sciences, USA) column (ID 20 mm × 50 cm), was used for further purification and the final polysaccharide fraction was lyophilized for further study

### Fourier Transform Infrared Spectroscopy Analysis

AAP1 mixed with potassium bromide (KBr) powder as KBr discs. A disc containing 2% (w/w) of finely ground powder of APP1 was prepared. The Fourier transform infrared spectroscopy (FT-IR) (Thermo Nicolet, Waltham, USA) was used to characterize the functional groups in the frequency range of 4000–400 cm^−1^.

### Animals and Experimental Design

A total of 36 specific-pathogen-free (SPF) male BALB/c mice (8 weeks old) were provided by the Vital River Laboratory Animal Technology Company (Beijing, China). Mice were kept under controlled environmental conditions at 25°C and with a 12-h light/dark cycle for one week. They were also fed *ad libitum* for this period. Then all mice were randomly divided into three groups (12 mice per group): the control, CTX, and AAP1 groups. Mice in the CTX and AAP1 groups were intraperitoneally injected with CTX at the dose of 80 mg/kg body weight (BW) on Day 7, 8, and 9 to induce immunosuppression (11), while the mice in the control group received an equal volume of 0.9% normal saline. In the next days, the mice in the AAP1 group were administered with 200 mg/kg BW of AAP1 by gavage once daily based on the data of preliminary experiments (**Table S1**). The control and CTX animals were gavaged with 200 μl normal saline per day at the same time, and the serum samples stored at −80°C for determining the levels of cytokines. Afterward, the cecal contents were then collected and stored at −80°C for gut microbiota and SCFAs analyses. The animal experimental protocol was approved by the Ethics Committee of the First Affiliated Hospital of Heilongjiang University of Chinese Medicine. Thymus and spleen indices were calculated as follows: 

Thymus or spleen organ index(mg/g) = organ weight (mg)/body weight (g)

### Determination of the Levels of Cytokines in Serum

The levels of interleukin-2 (IL-2), IL-4, IL-10, interferon-γ (IFN-γ), and tumor necrosis factor-α (TNF-α) in the mouse serum were assessed using commercial kits (Nanjing Jiancheng Bioengineering Institute, Nanjing, China) following the manufacturer’s instructions.

### Determination of the Levels of Cytokines in Serum

The relative expressions of Claudin-1, Occludin and ZO-1 were determined by real-time quantitative polymerase chain reaction (RT-qPCR), and GAPDH was used as an endogenous housekeeping gene. The primers (Comate Bioscience Co., Ltd, China) used in this study are shown in **Table S2**. Total RNA of colon tissue was extracted by RNAiso Plus (Takara, Dalian, China), cDNA was synthesized using the Transcriptor First Strand cDNA Synthesis Kit RNA (Roche, Germany), according to the manufacturer’s instructions. The PCR reactions were performed using Stormstar SybrGreen qPCR Master Mix (DBI Bioscience, Germany) on on the Bio-rad CFX96 real-time PCR System (Bio-rad, Foster City, CA, USA). The relative expressions of target genes were analyzed using the 2^-ΔΔCt^ calculation method.

### Gut Microbiota Analysis of Cecal Contents

The genomic DNA of cecal contents from all groups was extracted by a TIANamp Stool DNA Kit Total (Tiangen, Beijing, China) following the manufacturer’s instruction. After DNA isolations were examined, PCR primers (V3F: 5′-CCTACGGGNGGCWGCAG-3′; V4R: 5′-GACTACHVGGGTATCTAATCC-3′) were used to amplify the V3-V4 region of bacterial 16S rDNA. PCR products were sequenced using an Illumina Miseq (Illumina, Santiago, USA). Raw data were matched using FLASH software (V1.2.7) (28), and filtered with Quality Control software package. We filtered the low quality reads (More than 20% of the bases qualities are lower than 15), reads with adaptors and reads with unknown bases (N bases more than 5%) to get the clean reads (29, 30). The chimera sequences were discarded by using the UCHIME algorithm to achieve the clean tags (31). The tags were picked into distinct operational taxonomic units (OTUs) using Uparse software with a 3% divergence (32). They were sorted to different levels by comparing them to the GreenGene database using PyNAST software (Version 1.2) (33). The phylogenetic tree was constructed using MUSCLE software. Linear Discriminant Analysis Effect Size (LEfSe) was used to characterize potential biomarkers related to particular treatments with an effect size threshold of 2 (34). The raw data was deposited at Sequence Read Archive (SRA) in National Coalition Building Institute (NCBI) under accession no. PRJNA662015.

### Determination the Levels of SCFAs in Cecal Content

The levels of SCFAs (acetate, propionate, and butyrate) were determined as previously described (35). Briefly, cecal contents (50 mg) were homogenized with 0.3 mL purified water for 4 min at 45 Hz, ultrasonically treated for 5 min and centrifuged at 5000 g at 4°C for 15 min. The supernatant was filtered with 0.22 μm filter membrane, acidifed with 0.1 mL of 50% H_2_SO_4_, added with 0.4 mL of 2-Methylvaleric acid (internal standard) and subjected to gas chromatography-mass spectrometry (GC-MS) analysis using an Agilent 7890 gas chromatograph system coupled with an Agilent 5975C mass spectrometer (Agilent Technologies, Santa Clara, USA).

### Statistical Analysis

All data from this study are expressed as mean ± standard deviation (SD), and a minimum of three independent experiments were performed. The one-way analysis of variance (ANOVA) and the Duncan’s multiple range test was used to determine and compare statistical differences between means. Values of p < 0.05 were considered to be statistically significant.

## Results

### Isolation and Purification of EPS

**Figure 1:**
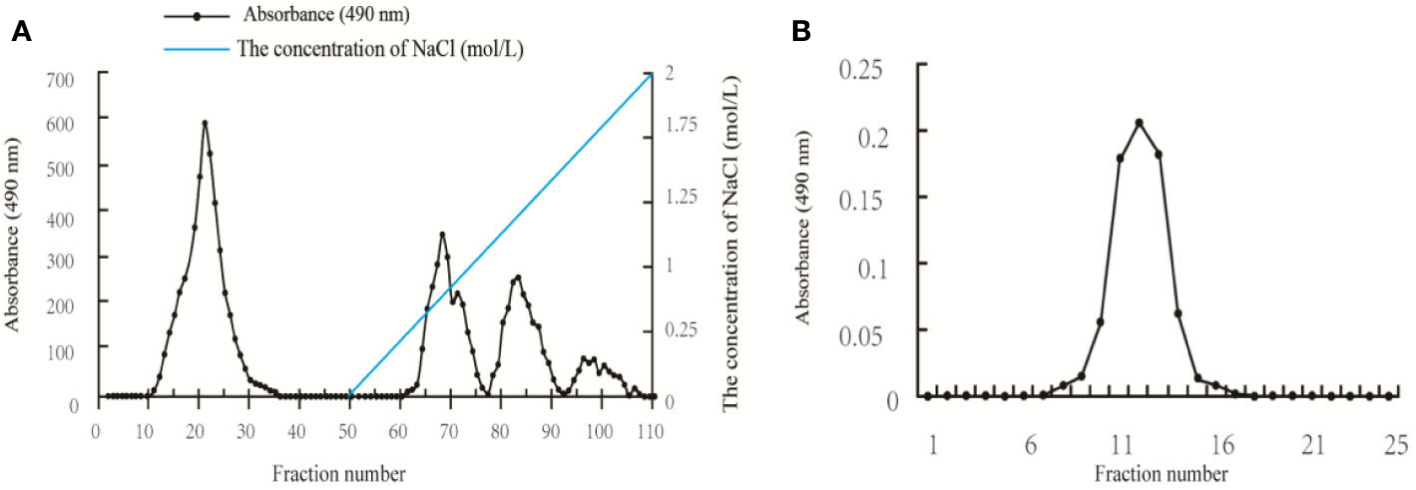
Elution profiles of the polysaccharides isolated from *Auricularia auricular*: **(A)** Elution profile of the *A. auricula* polysaccharides (AAP) on DEAE-52 column; and **(B)** Elution profile of AAP1 on Sephacryl S-400 column.

The crude polysaccharide isolated from *A. auricular* was first purified by an anion-exchange chromatography of DEAE-52 column, and four fractions (AAP1, AAP2, AAP3, and AAP4) were obtained (**Figure 1A**). Based on ionic group differences, AAP1 was neutral while AAP2, AAP3, and AAP4 were acidic. AAP1 was further purified because of a higher peak. Upon further purification, the AAP1 elution profile showed a single peak (**Figure 1B**), confirming that AAP1 is a homogenous polysaccharide.

### FT-IR Analysis of AAP1

**Figure 2:**
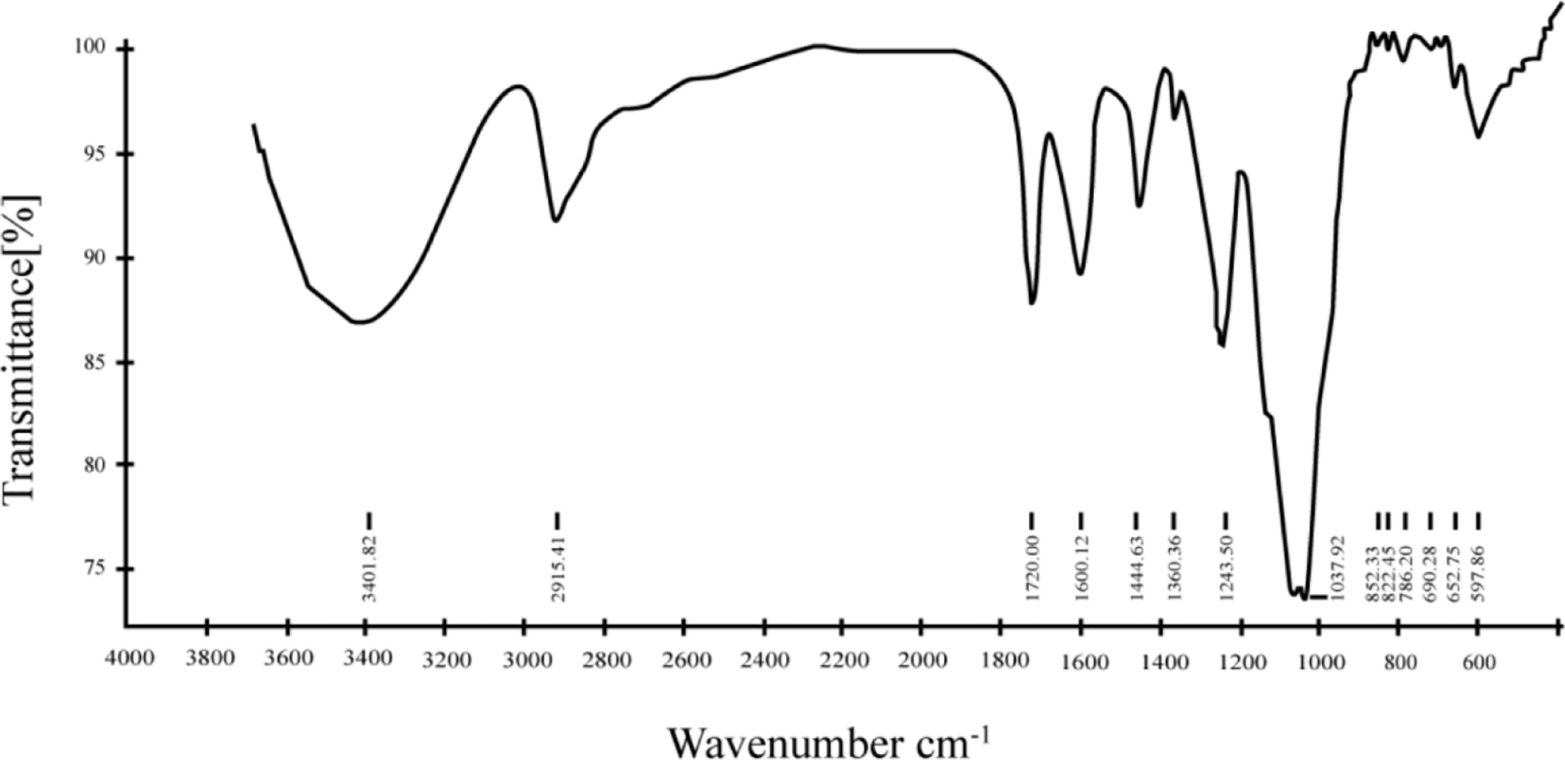
Fourier-transform infrared (FT-IR) spectra of AAP1 in the range of 400–4000 cm^-1^.

The FT-IR spectrum of AAP1 from 4,000 to 400 cm^-1^ is also reported in this study (**Figure 2**). The intensity and broad band at around 3,400 cm^−1^ in the spectrum of AAP1 can be attributed to the stretching vibration of the O-H group. The presence of the absorption around 2,920 cm^−1^ was assigned to the asymmetrical and symmetrical C−H stretching vibration. The peak at 1,640 cm^−1^ was the result of associated water bending vibration (36). Two bands at around 1,460 cm^-1^ and 1,250 cm^-1^ region were ascribed to skeletal C-O-C vibration (37). The presence of a bond around 1,100–1,010 cm^−1^ implied that the pyranoid ring was present in AAP1. The presence of bond around 850 cm^-1^ is α-type configuration sugar residue. A similar result was observed in polysaccharide of *A. auricular* reported by Morisaki et al. (38).

### Effects of AAP1 on Immune Organ Indexes 

As shown in **Table 1**, after treatment with CTX for three days, the thymus and spleen indexes of the model mice significantly reduced (p < 0.05) compared with those of the control group. Interestingly, significant improvements in these indices were observed in the AAP1 group, similar to the control group.

**Table 1:** Effect of AAP1 treatment on immune organ indexes.

Group	Thymus index (mg/g)	Spleen index (mg/g)
Control	0.95 ± 0.11*	5.47 ± 0.56*
CTX	0.71 ± 0.05	4.18 ± 0.33
AAP1	0.91 ± 0.61*	5.02 ± 0.24*

All data are expressed as mean ± SD. *p < 0.05: significantly different compared with the cyclophosphamide (CTX) group.

### Effects of AAP1 on Cytokine Production in Serum

**Figure 3:**
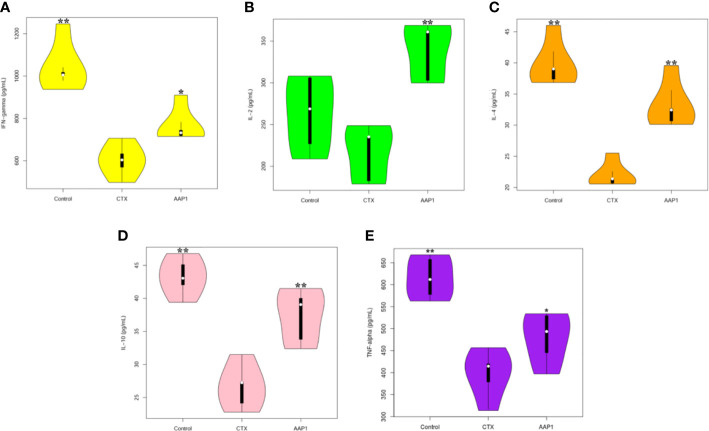
Effects of AAP1 on cytokine release in serum of cyclophosphamide (CTX)-treated mice. **(A)** interferon-γ (IFN-γ); **(B)** interleukin-2 (IL-2); **(C)** IL-4; **(D)** IL-10; and **(E)** tumor necrosis factor-α (TNF-α). All data are expressed as mean ± SD. *p < 0.05 and **p < 0.01: significantly different compared with the CTX group.

We used the ELISA protocol to evaluate the effects of AAP1 on the levels of IFN-γ, IL-2, IL-4, IL-10, and TNF-α in the serum (**Figure 3**). These five parameters in the CTX group were significantly lower than those of the control group (p < 0.05), indicating that CTX could inhibit immune activities. However, the levels of these cytokines were dramatically increased in CTX-treated mice after the AAP1 administration (p < 0.05). These results suggest that the AAP1 administration could reverse the immune-inhibitory activities of CTX by improving cytokine production.

### Effects of AAP1 on the Intestinal Barrier Function

**Figure 4:**
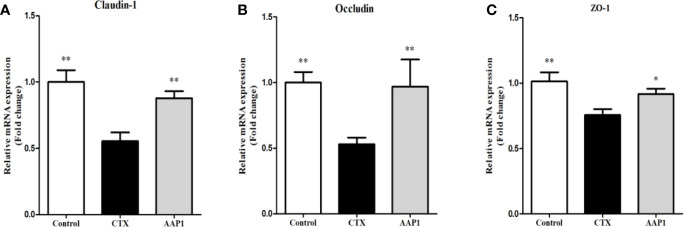
Effects of AAP1 on tight junction protein expression of colonic tissues of CTX-treated mice. **(A)** ZO-1, **(B)** Occludin, and **(C)** Claudin-1. All data are expressed as mean ± SD. *p < 0.05 and **p < 0.01: significantly different compared with the cyclophosphamide (CTX) group.

To estimate the effect of AAP1 on the colon intestinal barrier function, the mRNA expression of tight junctions (Claudin-1, Occludin and ZO-1) was determined. As shown as **Figure 4**, the mRNA levels of Claudin-1, Occludin and ZO-1 were significantly lower in the CTX group than that in the control group (p < 0.05). However, it was observed that the mRNA levels of Claudin-1, Occludin and ZO-1 were substantially up-regulated after the AAP1 administration (p < 0.05). These results suggest that AAP1 administration could improve the colon intestinal barrier function by regulating the mRNA expression of tight junctions.

### Effects of AAP1 on the Taxonomic Composition of the Gut Microbiota

**Figure 5:**
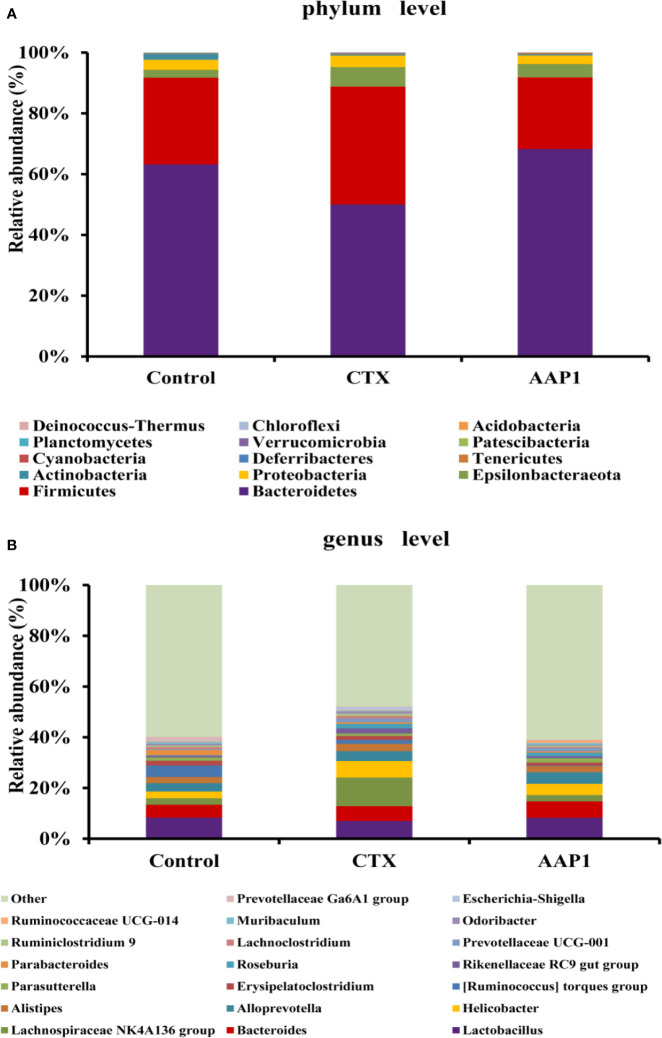
Effects of AAP1 on the gut microbiota at the level of phylum **(A)** and genus **(B)**.

The gut microbiota composition in all groups was analyzed using next-generation 16S rDNA sequencing. In all, 226,666 clean reads were obtained from cecal samples, and after a 97% similarity analysis was carried out, 300 ± 54 OTUs per sample were obtained. To further study the distinct taxonomic composition of the gut microbiota among all groups, the phylum and genus profiles were compared. As shown in **Figure 5A**, at the phylum level, the gut microbiota of all groups mainly consisted of Bacteroidetes and Firmicutes, which accounted for almost 90% of the total bacteria. The next predominant phyla were Proteobacteria and Epsilonbacteraeota. Compared with the control group, the increase in Firmicutes and Proteobacteria with a corresponding decrease in Bacteroidetes were observed in the CTX group. Interestingly, treatment with AAP1 resulted in a partial reversal of the CTX-induced gut dysbiosis.

At the genus level, individual bacteria groups were noticed (**Figure 5B**). The CTX group had higher abundances of *Odoribacter*, *Alistipes*, *Helicobacter*, *Escherichia-Shigella*, and *Oscillibacter* compared to the control group. However, these imbalances induced by CTX were reversed in part by AAP1 administration. Furthermore, the abundances of the SCFAs-producing microbiota *Bacteroides*, *Alloprevotella*, and *Blautia* and beneficial microbiota *Lactobacillus* remarkably increased in CTX-treated mice after AAP1 administration.

**Figure 6:**
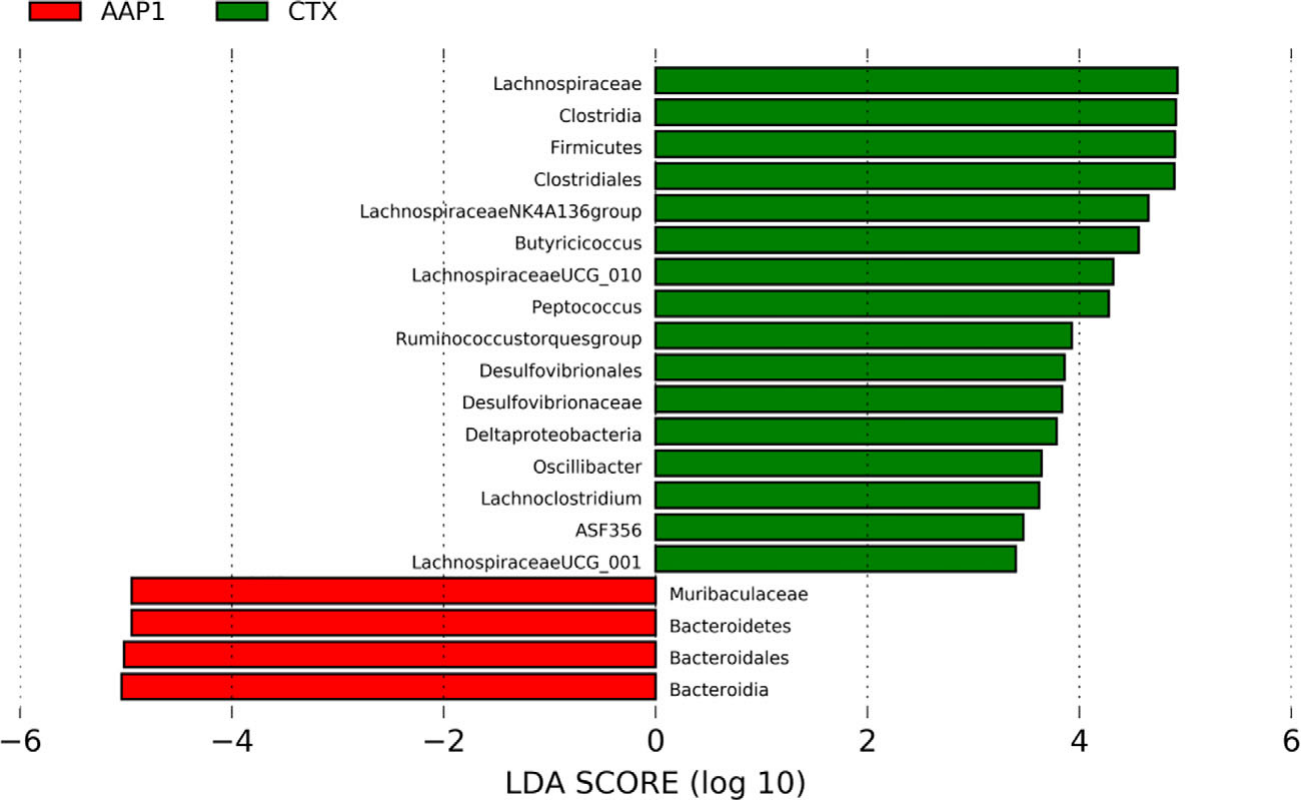
LEfSe analyses of gut microbiota regulation and immune response phylotypes. Phylotypes less than LDA 2 score are not reported.

In comparing the AAP1 and CTX groups, we used the LEfSe (LDA > 2) analysis was performed to identify the specific phylotypes in each group. This study identified 20 biomarkers involved in gut microbiota modulation and immune functions. Among them, four were higher, and 16 were lower in the AAP1 group than that in the CTX group (**Figure 6**). The results showed that the abundances of phylum Firmicutes and potentially pathogenic family Desulfovibrionaceae, genus *Peptoccus* and *Oscillibacter* were higher in the CTX-treated mice, whereas the beneficial phylum Bacteroidetes and family Muribaculaceae were lower in CTX-treated mice. These findings were in line with the above results.

### Effects of AAP1 on SCFAs Production

**Figure 7:**
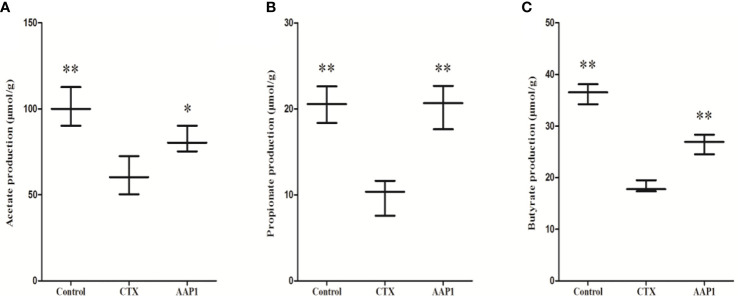
Determination of short-chain fatty acids (SCFAs) in cecal contents. **(A)** acetate; **(B)** propionate; and **(C)** butyrate. All data are expressed as mean ± SD. *p < 0.05 and **p < 0.01: significantly different compared with the cyclophosphamide (CTX) group.

SCFAs act as the critical bacterial metabolites, mainly composed of acetate, propionate, and butyrate in the intestine, which is beneficial for human health. This study showed that the contents of acetate, propionate and butyrate were significantly decreased in the CTX group when compared to the control group (**Figure 7**). Compared with the CTX group, AAP1 increased the contents of acetate, propionate and butyrate in CTX-treated mice. These findings indicated that AAP1 could enhance SCFAs production, which is in accordance with the increased abundances of SCFAs-producing microbiota.

## Discussion

The immune-inhibitory activities associated with the use of CTX in chemotherapy treatment can have profound impacts on health and wellbeing. The impaired host immune system induced by the chemotherapy can lead to increased incidences of secondary infections and immunodeficiency (39). Nowadays, among various health-promoting properties of natural polysaccharides, the enhancement of the immune response is of keen research interest. Recently, AAP has been widely studied by researchers due to its excellent immune-regulatory activity (25). However, little is known regarding the protective effects of AAP on CTX-induced immunosuppression as well as the mechanisms by which AAP ameliorates CTX-induced immunity disorders. Thus, the impact of AAP on the immunosuppression and the gut microbiota were investigated using an immunosuppressive model established by CTX in this study.

The thymus and spleen are two major building blocks of the human immune system. The thymus is recognized as the hub for T cell development, which in turn regulate the production of cytokines. T cells are known to play critical roles in a variety of immune responses against pathogens and some cancers. The spleen, on the other hand, is the site for the activation of biological substances facilitated by colonization of matured T and B cells. Thus, thymus and spleen indexes are known as the essential indicators reflecting immune functions (40). Our results showed that CTX decreased thymus and spleen indexes, indicating that the immunosuppressive model was successfully established. However, AAP1 treatment could significantly enhance these two indexes, implying that AAP1 had protective effects on the host immune system through imrpoving immune organs. These findings agreed with the results of polysaccharides from *Lycium barbarum*, which could effectively ameliorate these two indexes (13).

Cytokines are small-molecule soluble extracellular pleiotropic peptides or glycoproteins with multiple immune functions, including stimulating immune cells and specific non-immune cells (41). Regulatory T cell regulation plays an important role in the maintenance of immune adaptability to self-antigens and is involved in immune response modulation to ameliorate inflammatory processes. The population of TCRαβ+ CD4-/CD8- (double-negative, DN) T cells has attracted growing attention owing to their immune regulatory function (42, 43). As a pleiotropic cytokine, IFN-γ functions mainly in promoting protective immune responses and inhibits pathogen growth (44, 45). IL-2 function in inducing regulatory T cells, lowering disease severity in the host (46, 47). IL-4, produced by T helper 2 (TH_2_) cells, regulates allergic conditions and activates immune responses against external parasites (10, 48). The cytokine, IL-10, is produced by many cells of the adaptive immune system (49, 50). Although known widely for its pro-inflammatory activities, TNF-α is also used in hospitals to increase immune responses against tumors (51, 52). In our study, it was found that CTX inhibited the production of immune-related cytokines (IFN-γ, IL-2, IL-4, IL-10, and TNF-α), indicating that CTX is a potent immunosuppressive agent. Whereas, AAP1 treatment could reverse this poor situation. Findings from earlier reports have suggested that a number of polysaccharides can lower CTX-induced immunodeficiency by upregulating cytokine production. The polysaccharides from *Sargassum fusiforme* could ameliorate immunocompromised mice induced CTX by increasing the generation of IL-2, IL-6, and IFN-γ (53). The secretion of cytokines TNF-α and IL-2 was enhanced in CTX-treated mice after administration with polysaccharides from *Ganoderma atrum* (54). The underlying mechanisms, however, appear rather complicated. On the one hand, polysaccharides can induce signaling within immune cells through the Toll-like receptor (TLR)-2 and TLR-4, which in turn induce cytokine secretion (55). Tight junction proteins like Claudin-1, Occludin and ZO-1 play an important role in maintaining the integrity of tight junctions. The abnormal expression of Claudin-1 is a common feature of various diseases, which can reflect intestinal barrier damaged and restored (56). Occludin was involved in regulating 'leak pathway', whose decreased expression and abnormal distribution result in increased permeability of monolayer intestinal epithelial cells (57). ZO-1 is one of the important components of the tight junction, which attaches Claudin and Occludin proteins to the cytoskeletonis, and often used as an indicator of intestinal inbarrier function (58). Our present results showed that AAP1 could significantly increase the level of Claudin-1, Occludin and ZO-1, suggesting that AAP1 could effectively restore intestinal barrier functions. On the other hand, various polysaccharides could regulate the gut microbiota composition altered by CTX, thereby improving the host immunity. In this study, effects of AAP1 on the gut microbiota composition in CTX-treated mice were investigated by 16S rDNA V3–V4 region sequencing technology.

In our study, at the phylum level, the CTX group exhibited striking increases in the abundances of Firmicutes and Proteobacteria but decrease in Bacteroidetes when compared to those of the control group. The increased the ratio of Firmicutes/Bacteroidetes was in line with previous studies (6, 59). In additon, similar changes in the gut microbiota were also found in patients after surgical ileostomy, small bowel transplantation and animal with colon cancer and deficient immune (60–62). These findings indicated that the increased the ratio of Firmicutes/Bacteroidetes could promote the development of immunosuppression. After the AAP1 adminstraion, Bacteroidetes was significantly increased and Firmicutes and Proteobacteria were significantly decreased relative to the CTX group. Similarly, the consumption of polysaccharides from *A. auricular* could reduce in the Firmicutes/Bacteroidetes ratio (24). As reported, while Bacteroidetes use multiple enzyme combinations in breaking down polysaccharides (63), Firmicutes are more specialized in polysaccharide metabolism. Noteworthy, the genome functionality of the former is significantly higher than the latter (64, 65). So far, several natural polysaccharides have shown a direct relationship with Bacteroidetes proliferation and an inverse proportion to Proteobacteria growth (11, 66, 67). This suggested that the immuno-enhancing potential of AAP1 was associated with regulating the abundances of these main phyla.

At the genus level, the abundances of *Odoribacter*, *Alistipes*, *Helicobacter*, *Escherichia-Shigella*, and *Oscillibacter* were enriched in the CTX group when compared to the control group. However, the AAP1 administration showed a reversal to normalcy. *Helicobacter* is a Gram-negative microorganism and can result in pro-inflammatory effects (68). In contrast, Sun et al. reported that the abundance of *Helicobacter* was reduced in the CTX group (14). However, an elevation in *Helicobacter* has been reported in the CTX group and polysaccharides from purple sweet potato could lessen it (11). *Escherichia-Shigella* has been implicated in the onset of inflammatory bowel disease as well as other gastrointestinal disorders (69). Over-representation of *Alistipes* was not only observed in the CTX-treated mice (6), but also in old mice with the low ability of immune regulation (70). It has been demonstrated that *Odoribacter* is often associated with the human immunodeficiency virus (HIV) epidemic, which caused immunocompromised hosts (71).

Apart from the aforementioned genera, the relative abundances of *Bacteroides*, *Alloprevotella*, *Blautia*, and *Lactobacillus* were increased in CTX-treated mice after administration with AAP1. *Lactobacillus* is one of the most common probiotic species and is important for the maintenance of healthy homeostasis (72). *Lactobacillus* administration can have the potential for improving immune reactivity. These were accompanied by enhancing CD^+^ T cell-dendritic cell interactions, lymphocyte proliferation, and cytokine secretion (IL-1β, IL-6, IL-10, IL-12, IFN-γ, and TNF-α) in mucosal immunity in a recent study (73–76). *Lactobacillus* can also produce lactic acid, which can be used as a precursor for butyric acid synthesis (77). *Bacteroides*, *Alloprevotella*, *Blautia* are well-known SCFAs-producing microbiota. SCFAs, including acetate, propionate, and butyrate are the primary metabolites produced by the gut microbiota and play important roles in maintaining the normal function of the immune system (78–80). More so, the increased proliferation of SCFAs-producing microbes gives a corresponding increase in the cecal SCFA levels. In this study, we also found that AAP1 could obviously enhanced the production of SCFAs. Polysaccharides can influence the immune system as a result of microbial products such as SCFAs, which may interact with immune cells and enterocytes in regulating and maintaining the normal function of the innate and adaptive immune system (78, 79).

In particular, butyrate has been recently reported to have specific immune-modulatory functions by maintaining intestinal homeostasis through anti-inflammatory actions (81). SCFAs, through G-protein coupled receptors (GPR41, GPR43, and GPR109A) can also induce immune protection (82). These receptors broadly function in the secretion of chemokines and cytokines, which are vital defense mechanisms (83). It follows, therefore, that SCFAs can induce epithelial cells to not only maintain intestinal homeostasis but also boost immunity levels. This postulation agrees with the findings of this study. Several polysaccharides have been demonstrated to be efficacious in immune-protective function on CTX-treated mice, which are achieved through acting as a substrate of gut microbiota to produce SCFAs (11, 13, 14). The present study indicated that AAP1 might contribute to its immunostimulatory effect on the immunosuppressed mice by modulating the gut microbiota and their metabolites-SCFAs.

## Conclusion

This study showed that the AAP1 administration had immune-protective effects on CTX-treated mice *in vivo* by significantly enhancing immune organ indexes and stimulating the production of cytokines in the serum, including IFN-γ, IL-2, IL-4, IL-10, and TNF-α, and up-regulated the mRNA levels of Claudin-1, Occludin, and ZO-1. Furthermore, AAP1 could regulate the composition of gut microbiota and enhance the synthesis of SCFAs in immunosuppressed mice. Our findings suggest that the AAP1 can be used as gut microbiota regulators with health-promoting potentials as well as an immunomodulator in foods and drugs.

## Data Availability Statement

The original contributions presented in the study are included in the article/**Supplementary Material**. Further inquiries can be directed to the corresponding author.

## Ethics Statement

The animal study was reviewed and approved by Ethics Committee of the first Affiliated Hospital of Heilongjiang University of Chinese Medicine.

## Author Contributions

ZD and XK conceived the study and designed the project. XK, WD, DL, and XT performed the experiment and analyzed the data. XK drafted the manuscript. ZD revised the manuscript. All authors contributed to the article and approved the submitted version.

## Funding

Present research work was financially supported by grants from Natural Science Foundation of Heilongjiang Province (ZD2020C010), Pre-research Project of Heilongjiang Academy of Sciences (YY2020SW03), President Fund Project of Heilongjiang Academy of Sciences (YZ2020SW01) and 2019 Guangxi university young and middle-aged teachers' basic scientific research ability improvement project (2019KY0711).

## Conflict of Interest

The authors declare that the research was conducted in the absence of any commercial or financial relationships that could be construed as a potential conflict of interest.
