# Supplementary material for: Effects of Polysaccharides From Auricularia auricula on the Immuno-Stimulatory Activity and Gut Microbiota in Immunosuppressed Mice Induced by Cyclophosphamide
Source: Front Immunol. 2020 Nov 6;11:595700. doi: 10.3389/fimmu.2020.595700 (PMC7681245; doi:10.3389/fimmu.2020.595700)
Supplement: Supplementary file 1 [file DataSheet_1.docx]

**Table S1** Effects of different doses of AAP1 on cytokine release in serum of CTX-treated mice.

|  | IFN-γ (pg/mL) | IL-2 (pg/mL) | IL-4 (pg/mL) | IL-10 (pg/mL) | TNF-α(pg/mL) |
| --- | --- | --- | --- | --- | --- |
| Control | 986.86 ± 43.34 ^a^ | 261.2 ± 49.04 ^b,c^ | 38.53 ± 0.98 ^a^ | 42.18 ± 2.85 ^a^ | 619.12 ± 45.61 ^a^ |
| CTX | 566.98 ± 67.51 ^d^ | 172.46 ± 28.73 ^d^ | 20.89 ± 0.43 ^d^ | 24.76 ± 2.36 ^d^ | 404.27 ± 22.43 ^c^ |
| APP1-50 | 622.22 ± 25.63 ^c,d^ | 247.01 ± 35.15 ^b,c^ | 22.67 ± 2.15 ^d^ | 27.33 ± 2.95 ^c,d^ | 436.36 ± 19.51 ^b,c^ |
| AAP1-100 | 676.91 ± 25.17 ^b,c^ | 299.54 ± 39.85 ^a,b^ | 25.56 ± 0.75 ^c^ | 30.60 ± 1.34 ^b,c^ | 469.91 ± 34.38 ^b^ |
| APP1-200 | 725.93 ± 15.88 ^b^ | 343.66 ± 38.12 ^a^ | 31.92 ± 1.08 ^b^ | 35.39 ± 4.06 ^b^ | 489.34 ± 42.24 ^b^ |

APP1-50, the mice in the group were administered with 50 mg/kg BW of AAP1 by gavage once daily; AAP1-100, the mice in the group were administered with 100 mg/kg BW of AAP1 by gavage once daily; AAP1-200, the mice in the group were administered with 200 mg/kg BW of AAP1 by gavage once daily. The mean values in the same column with different letters mean significantly different (p < 0.05) by a Duncan’s multiple range test.

**Table S2** Primer sequences of mRNA for RT-qPCR.

| Genes | Primer sequence (5'-3') |
| --- | --- |
| GAPDH | F: ATGGGAAGCTTGTCATCAACG |
|  | R: AAGACACCAGTAGACTCCACG |
| ZO-1 | F: GGAGCAGGCTTTGGAGGAG |
|  | R: CCACCGTCCGCATAAACATC |
| Occludin | F: GCCCCTCTTTCCTTAGGCG |
|  | R: AAGATAAGCGAACCTGCCGA |
| Claudin-1 | F: TCTACGAGGGACTGTGGATG |
|  | R: TCAGATTCAGCTAGGAGTCG |
